# Reliability and validity of the international physical activity questionnaire in the Nord-Trøndelag health study (HUNT) population of men

**DOI:** 10.1186/1471-2288-8-63

**Published:** 2008-10-09

**Authors:** Nanna Kurtze, Vegar Rangul, Bo-Egil Hustvedt

**Affiliations:** 1 HUNT Research Centre, Faculty of Medicine, Department of Public Health and General Practice, Norwegian University of Science and Technology, Trondheim, Norway; 2 Department of Health Science, Nord-Trøndelag University College, Levanger, Norway; 3 Institute of Basic Medical Sciences, Department of Nutrition, University of Oslo, Norway

## Abstract

**Background:**

There is no standardized method for the assessment of physical activity (PA). Therefore it is important to investigate the validity and comparability of different measures. The International Physical Activity Questionnaire (IPAQ) has been developed as an instrument for cross-national assessment of PA and has been validated in 12 countries. These instruments have acceptable measurement properties for monitoring population levels of PA among 18–65 year-old adults in diverse settings. However, there are some concerns that IPAQ may over-report PA.

The purpose of this study is to evaluate the reliability and validity of IPAQ, short version, last 7-days in the Nord-Trøndelag Health Study (HUNT) population of men.

**Methods:**

The questionnaire was administered twice to a random sample of 108 men aged 20–39 and validity by comparing results with VO_2max _and ActiReg, an instrument that measures PA and energy expenditure (EE). ActiReg discriminates between the body positions: stand, sit, bend forward and lie and also registers if there is motion or not in each of them or both.

**Results:**

Our results for reliability of the IPAQ short version were good for vigorous and fair for moderate activities. Intraclass correlations ranged from a low of 0.30 for moderate activity hours, to a high of 0.80 for sitting hours. Concerning validity, our results suggest that total IPAQ vigorous PA was a moderately good measure of vigorous activity, having moderately strong, significant correlations with VO_2max_, r = 0.41 (p ≤ 0.01), but correlated not with metabolic equivalent (METs) values of 6 or more measured with ActiReg. Only total IPAQ walking was fair correlated with METs 1–3 and METs 3–6, respectively r = -0.27 and 0.26 (p ≤ 0.05). The index for IPAQ sitting hours per week was moderate correlated with METs values of 1–3 and negatively correlated with METs values of 3–6. Classification of PA in three levels (low, moderate and high) correlated also most strongly with VO_2max _(0.31 p ≤ 0.01) and METs 3–6 and METs 1–3 from ActiReg (r = 0.32 and -0.31, p ≤ 0.01). Classification of BMI in three levels (normal, overweight and obese) correlated most strongly negative with VO_2max _(-0.42 p ≤ 0.01) and MJ from ActiReg (r = 0.31 p ≤ 0.01).

**Conclusion:**

Our results indicate that IPAQ short version for men has acceptable reliability and criterion validity for vigorous activity and sitting. Walking has moderate reliability. Only the IPAQ for walking had a fair correlation with METs 6+. The questions about moderate activity had fair reproducibility and correlated poorly with most comparison measures.

## Background

Questionnaires are typically used in epidemiologic studies to assess physical activity (PA) levels, but the absence of widely accepted standardized methods and the use of different PA measures hinders national and international comparisons. However, the International Physical Activity Questionnaire (IPAQ) has been developed as an instrument for cross-national assessment of PA and has now been validated in 12 countries [1]. These instruments have acceptable measurement properties for monitoring population levels of PA among 18–65 years old adults in diverse settings. Long and short versions of the IPAQ are available which can be administered by telephone interview or self-administration. Two reference periods can be investigated, either the "last 7 days or "usual week". Over-reporting of PA, especially of time and intensity [2], may be a problem in self-reported assessments, and there are some concerns that IPAQ may share this tendency.

Validity and reliability of the IPAQ has been addressed in several studies. One study has addressed possible over-reporting in IPAQ, using the short last 7 day, telephone interview (IPAQ-S7T) [3]. In the European Physical Activity Surveillance System (EUPASS) researchers have compared several PA measures, including IPAQ, in a time series in eight countries of the European Union, highlighting methodological implications of the IPAQ (computer-aided telephone interviewing) [4]. In another study, Rütten et al. [5] concluded that more research is needed to further investigate and improve the quality of the IPAQ (telephone interviewing). A Chinese version of IPAQ (long and short version) appeared to have acceptable reliability and validity, compared to other PA instruments used in various large epidemiological studies, although the short version underestimated the energy expenditure (EE) of total and moderate PA [6]. Hallal & Victora [7] reported that the short version may systematically underestimate PA levels in Brazilian adults. A recent study suggested IPAQ is a reliable and valid measurement of total PA in a Chinese population, however the sub-components of total activity were less valid and reliable, (short, last 7 days) [8].

To our knowledge, when this study started, no one had yet criterion-validated IPAQ against physical fitness measures even though IPAQ was designed to measure particularly activities related to cardiorespiratory fitness [1]. However, Fogelholm [9] has later published a study comparing the IPAQ against VO_2max_, measured by submaximal ergometer cycle testing (self-administered IPAQ short format, last 7 days, S7S). A recent study by Ekelund et al. [10] also examined the short IPAQ, last 7 days version in Swedish adults, claiming their study to be the first to evaluate the absolute time spent in moderate and vigorous PA from the IPAQ questionnaire.

The purpose of this study is to evaluate the reliability and validity of the IPAQ short version questionnaire in a Norwegian sample of the Nord-Trøndelag Health Study (HUNT) population of men. It is unique in that we assess validity by directly comparing questionnaire results with direct measurements of PA and energy expenditure (EE) assessed with a position and motion sensor, ActiReg, as well as indirectly by comparing with VO_2max_.

## Methods

### Subjects

For this study 108 men, aged 20–39 were randomly selected from the population of Levanger and Verdal, two of the communities included in the Nord-Trøndelag Health Study (HUNT). They were selected with the goal to be representative of the current population of young adult men in the area. 250 were randomly selected by a statistical service and invited; 108 accepted. With the sample size of 100, the study had an estimated 88% power to detect a correlation of 0.3 between the score from the IPAQ questionnaire and VO_2max_. The population of Nord-Trøndelag is stable with sex- and age distributions similar to those of Norway as a whole, but with somewhat lower levels of education and income compared to national averages.

An invitation letter was mailed with a pre-addressed, postage paid envelope, which was to be completed and returned; participation was voluntary. Height was measured without shoes with a wall-mounted tape measure, and weight with a laboratory scale (Heine Professional, 7800) at the test laboratory of Nord-Trøndelag University College. Body mass index (BMI) was computed as weight in kilograms divided by height in meters squared. We also recoded BMI in a categorical score, classified into three levels ("normal" (< 25 kg/m^2^), "overweight" (25–29.99) and "obese" (≥30 kg/m^2^).

The study was approved by The Norwegian Data Inspectorate Board (IRB) and each subject gave his written informed consent prior to participation in the study. Further, the Regional Committee for Ethics in Medical Research recommended the protocol.

### Survey measures of IPAQ short version, self-administered last 7 days (IPAQ-S7S)

PA was assessed using the IPAQ-S7S translated into Norwegian by The Norwegian Directorate of Health and Services. We used the instructions given in the IPAQ manual for reliability and validity, which is detailed elsewhere [1].

To sum up the single indicators to an overall indicator of PA-related EE (Metabolic equivalent, MET min ^-1^) is a major goal of the IPAQ instruments. We used the recommended, following MET estimates of IPAQ: Vigorous PA = 8 METs, moderate PA = 4 METs, walking on average = 3.3 METs. For calculating the overall METs PA, each category was multiplied with its special MET estimate value (we call it IPAQ METs). We also did some recoding: if someone said they "never" walked or walked "0" days per week, then hours per day and minutes per day were coded as "0".

We also used the recommended categorical score, three levels of PA (low, moderate and high) as proposed in IPAQ Scoring Protocol (short form). Low activity represented individuals who do not meet the criteria for moderate and vigorous intensity categories (< 599 MET-min/week). Moderate activity represented moderate – or vigorous -intensity activities achieving a minimum of at least 600 Met-min/week. High activity represented achieving a minimum of a least 3000 Met-min/week.

Before the VO_2max _test, participants completed the self-administered IPAQ short version. This version consists of 7 questions addressing PA, over the last 7 days in all context of everyday life. Addressing days, hours and minutes per week in vigorous PA, moderate activity, walking and sitting during a weekday. For each intensity group we recoded a total IPAQ by computing an index of days, hours and minutes. These questions from IPAQ we also calculated METs and hours per week in vigorous and moderate activities, walking and sitting, as described by Craig et al. [1] and already mentioned.

### Reproducibility

Reliability was evaluated by the asking each subject to complete the questionnaire one week after first taking it, using a test-retest design.

### Validity

In the absence of a true "gold standard" and as PA is a multidimensional exposure, we evaluated validity by comparing the PA data from the self-reported IPAQ with several measures related to PA: VO_2max _and five measures of PA assessed with ActiReg – Megajoule (MJ) per day, hours at metabolic equivalent (METs) > 6, hours at METs 3–6, hours at METs 1–3 average per day and physical activity level (PAL), defined as total EE divided by basal metabolic rate (BMR) [11]. As many others studies, we used VO_2max _[12,13] which reflects fitness, as an indicator of PA-related fitness or aerobic training. A correlation between VO_2max _and PA assessed using the questionnaire would suggest that the questionnaire was measuring aspects of PA, such as aerobic training-related fitness level. The measures obtained from ActiReg reflect different aspects of PA, measured more directly.

We have used the first test of IPAQ in comparison against VO_2max _and ActiReg, which means that the participants were not familiar with the questionnaire and they were not given a "training round".

### Testing for Maximal oxygen uptake (VO_2max_)

For measuring VO_2max _(ml·kg^-1^·min^-1^) Metamax II (Cortex Biophysic GmbH, Leipzig, Germany), a metabolic analyzer was used (serial no. MII 63 229 901). The instrument was used stationary at the physiological test laboratory at Nord-Trøndelag University College. It recorded and displayed data at 10 s averages which was transferred to a PC using the programme Cortex Metasoft. The Metamax II oxygen analysers has been examined in a study using the Douglas bag technique as the control method [14]. The instrument has built-in sensors for O_2_, CO_2, _a barometer and a thermometer which measure the flow of the breathed air by means of a turbine flow meter attached to the breathing mask or mouthpiece. The instrument was calibrated against ambient air and a commercial gas of known concentrations of O_2 _(16.00%) and CO_2_(4.00%) in the morning before the start of each experiment. The concentration of O_2 _and CO_2 _of room air was read and the flow transducer was calibrated using a 3 -L high-precision calibration syringe (Calibration syringe D, SensorMedics, Yorba Linda, CA) before testing a new subject.

The subjects were instructed not to eat or smoke at least 2 hours before the test. Water could be taken as needed at any time. No unusual physical activity efforts should have been performed at least 12 hours before testing, and subjects were to dress appropriately for exercise, especially with regard to footwear.

Before the VO_2max _test the participants also signed a statement that they were healthy and fit for the VO_2 _max test on treadmill; all 108 participants declared themselves fit.

A maximal treadmill exercise test was performed to test VO_2max _in accordance with recommendations [15]. The speed was increased gradually and the incline was steady at 5%. Before the test, subjects had warmed up for 15 min at 5% gradient and individual speed. After the warm up the subjects prepared to start the test after 2–3 minutes break. The test started with 2 minutes of exercise intensity corresponding to approximately 60% of VO_2max_. This intensity was estimated during the warm up. After 2 minutes the treadmill speed increased gradually during 1 minute to a level that brought the subject close to exhaustion in approximately 3–4 minutes. This workload should have been kept up for 3–5 minutes. We asked subjects to continue for 4 minutes. After 2 minutes the respiratory quotient (RQ) should be at least 1.00. If it was lower, the speed was increased, if the RQ was greater than 1.20 we had to reduce the speed. During the last 2 minutes the workload could not increase, but before that it's possible to increase the workload up to 10%. During the test, RQ was estimated to control for satisfactory workload. When the O_2 _uptake showed no further increase or increased only slightly with a further increase in the treadmill speed and if the RQ ≥ 1.05 the test was considered successful. Otherwise the run was continued until a levelling-off was seen or until exhaustion. A subject's VO_2max _was taken as the median of the three successive highest O_2 _registrations.

### ActiReg for measurement of PA and EE

In order to measure total EE, intensity of expenditure and time spent at various intensity level a relatively new instrument, ActiReg (PreMed AS, Oslo, Norway) was employed. ActiReg is an electromechanical device, which records the main body positions (stand, sit, bent forward and lie) together with motion of the trunk and/or one leg each second [16]. The position (tilt switches) and motion sensors are fixed to plastic brackets. During registration the subjects wear ActiReg in a belt and the sensors are connected to the box with thin cables. The brackets are attached by medical tape to the subject's chest (on sternum) and on the front of the right thigh approximately midway between the knee and the hip. The tilt switches are oriented so that they will be in the vertical position when the subject is standing.

A dedicated computer program (ActiCalc32) calculates EE and activity pattern from the collected information and calibration data [16]. The calculation model used by ActiCalc32 are based on the estimated cost of the actual body position and activity expressed as physical activity ratios (PAR) values (i.e. EE/RMR) combined with the number of position changes within each minute. PAR values used by the ActiCalc32 program during calculation of EE are published reference values for people with normal body weight [17]. The ActiReg system has been validated both against doubly labeled water and indirect calorimetry [16]. More details about the method are published elsewhere [16]. Thus each minute of the registration period is characterized according to its estimated PAR-value which can then be categorized into light, moderate and vigorous activity, such as METs 1–3, METs 3–6 and METs 6+.

After VO_2max _testing participants were instructed how to wear ActiReg. In addition participants received an illustration with written instructions and a memory list about the use of ActiReg. They wore ActiReg for 7 consecutive days, all hours except while sleeping. For 15 people, fewer than 7 days were available, and they were excluded. We base results only on the days with measurements.

### Data analysis

Statistical analyses were performed with SPSS, Inc., Chicago IL, version 15.0. Sample Power 2 was used to estimate the required sample size. To evaluate reliability, we calculated single measure intraclass correlation coefficients (ICC). A 95% confidence interval (CI) was used to describe the variety/difference in the ICCs. To assess validity, we used Spearman correlation coefficients to measure the association of the questionnaire responses with VO_2max _and ActiReg results. We also used Pearson correlation coefficients for comparison. We used ordinary least squares regression to assess and to adjust for possible covariates, but present results only from the Spearman correlation analyses since our results and conclusions were similar with other methods. Agreement between self-reported time spent in total IPAQ moderate and total IPAQ vigorous PA and measured time spent at the same intensity level by ActiReg was assessed with an Bland-Alman technique [18]. We plotted the difference between the criterion-measured (by ActiReg) time spent in METs 3–6 and METs 6+ and self-reported total IPAQ moderate and total IPAQ vigorous PA against the criterion.

## Results

As shown in Table 1, the mean age of study subjects (N = 108) is 32.4 years and the means for weight, height and BMI are 85.5 kg, 180.2 cm and 26.3, respectively. Sixteen in the group who were 20–29 years of age and 45 in the group who were 30–39 years of age were classified as preobese (BMI 25.0–29.9), based on WHO's classification of BMI; four and nine in each age group, were in Obesity class 1 (BMI 30.0–34.9).

**Table 1:** Physical characteristics, classification of BMI, VO_2max _(ml·kg^-1^·min^-1^) and normal values of maximal oxygen uptake at the age of 20–39 and in the validation study

**Physical characteristics of subjects 20–39 year (N = 108)**								
	Age (yr)		Weight (kg)		Height (cm)		BMI	
	Mean	SD	Mean	SD	Mean	SD	Mean	SD
Male	32.4	5.24	85.5	12.20	180.2	7.88	26.3	3.16
								
**Classification of BMI among subjects**								

Age, yr	< 18.50	18.50–24.99	25.00–29.99	30.00–34.9				
20–29	1	10	16	4				
30–39		23	45	9				
								
**Subjects characteristics of VO_2max _(ml·kg^-1^·min^-1^)**								

Age, yr (N)	Low (N)	Somewhat low (N)	Average (N)	High (N)	Very high (N)			
20–29 (31)	38 (3)	39–43 (10)	44–51 (13)	52–56 (4)	57 (1)			
30–39 (77)	34 (2)	35–39 (8)	40–47 (40)	48–51 (12)	52 (15)			
								
**Normal values of maximal oxygen uptake, age of 20–39 and in the validation study**								

Normal values			Validation study					
Age, yr, men	Mean	SD	Age, yr, men	Mean	SD			
20–29 (ml·kg^-1^·min^-1^)	43	7.2	20–29 (ml·kg^-1^·min^-1^)	45.8	6.39			
METs	12		METs	12.7				
30–39 (ml·kg^-1^·min^-1^)	42	7.0	30–39 (ml·kg^-1^·min^-1^)	46.1	6.22			
METs	12		METs	12.7				

Values are expressed as mean and standard deviation (SD). MET indicate metabolic equivalent 3.5 ml·kg^-1^·min^-1^.

Maximal oxygen uptakes were generally comparable to age-specific "normal" values [19], although the age-specific averages for men in this study were slightly higher. Ten in the group 20–29 years of age, and eight in the group 30–39 years of age had a somewhat low oxygen consumption, whereas four and twelve, respectively, had a high oxygen consumption in relation to classification of VO_2max _[20],

Table 2 presents means and standard deviations of selected measures of physical activity and fitness for subjects in this study. The mean (standard deviation) VO_2max _was 45.99 (6.24) and the mean (standard deviation) daily EE was about 12.7 MJ (1.82).

**Table 2:** Means and standard deviations for responses to physical activity questionnaire (IPAQ short version, last 7 days) and criterion measures

Measures	N	Mean	SD
Vigorous activity days/week	107	1.78	1.80
Vigorous activity hours/day	104	1.29	1.86
Moderate activity days/week	106	2.18	2.04
Moderate activity hours/day	93	1.27	1.67
Walking 10 min. days/week	105	3.55	2.71
Walking hours/day	95	1.11	2.22
Sitting hours/day	92	5.91	3.5
			
Total IPAQ METs min/week	84	4157.07	6095.86
			
3 PA categories	84	2.12	0.75
			
BMI	108	26.3	3.16
BMI 3 categories	108	1.81	0.63
			
VO_2max _(mL·kg^-1^·min^-1^)	108	45.99	6.24
			
ActiReg			
EE (MJ^1)^)	93	12.71	1.82
PAL^2)^	93	1.68	0.2
METs1–3^3)^	93	20.97	1.34
METs3–6^3)^	93	2.69	1.26
METs 6+^3)^	93	0.27	0.26

^1) ^average EE (MJ) per day

^2) ^PAL = average physical activity level in 7 days

^3) ^METs = average hours per day

3 PA categories = Classification of physical activity in three levels; "low", "moderate" and "high"

BMI (Body Mass Index) 3 categories = Classification of BMI in three levels ; "normal" (< 25 kg/m^2^), "overweight" (25–29.99) and "obese" (≥30 kg/m^2^)

### Questionnaire Reliability

Table 3 gives ICC for the IPAQ questionnaires (N = 108). Correlations ranged from a low of 0.30 for moderate activity hours, to a high of 0.80 for sitting hours.

**Table 3:** Test re-test reliability based on intraclass correlation coefficients (ICC) for the IPAQ (short version, last 7 days) questions (N = 108)

Questionnaire	N = 108	
	
	ICC	95% CI
IPAQ		
Vigorous activity (days per week)	0.61	0.48 – 0.72
Vigorous activity (hours per day)	0.62	0.47 – 0.73
Moderate activity (days per week)	0.34	0.16 – 0.51
Moderate activity (hours per day)	0.3	0.09 – 0.49
Walking (days per week)	0.56	0.41 – 0.68
Walking (hours per day)	0.42	0.23 – 0.59
Sitting (hours per day)	0.8	0.70 – 0.87

ICC = Single measure intraclass correlation coefficient

### Validity

#### Associations with ActiReg and VO_2max_

As can be seen in Table 4, VO_2max _which is sometimes referred to as a criterion measure of fitness, tended to increase as the hours with vigorous activity increased, up to 4–6 hours per week. However, the trends were not monotonic, possibly reflecting the small number of subjects in some categories. However, those with 4–6 hours of vigorous PA had the highest mean of VO_2max _(49.2). When VO_2max _data were categorized into 2 or 3 groups, there was a clear trend showing a higher activity levels with increased fitness level. However, the differences were not significant, due to the nature of the activity scores which have zero values. This leads to high SD scores. The trends for other measures are less consistent.

**Table 4:** Distribution of criterion measures based on VO_2max _and ActiReg by IPAQ (short version, last 7 days) questionnaire responses

Mean (SD) for Each Factor				
Measures	VO_2max_	METs 1–3^2)^	METs 3–6^2)^	METs 6+^2)^

Vigorous activity hours/week^1)^				

1	43.2 (4.5)	20.96 (1.50)	2.67 (1.41)	0.24 (0.28)
2-3	49.2 (6.1)	20.92 (1.23)	2.65 (1.09)	0.35 (0.27)
4-6	49.2 (5.5)	21.21 (1.00)	2.51 (1.05)	0.27 (0.19)
6+	48.4 (6.9)	20.73 (1.33)	3.02 (1.27)	0.26 (0.29)

Moderate activity hours/week^1)^				

1	44.7 (5.9)	21.08 (1.52)	2.57 (1.46)	0.26 (0.29)
2-3	48.3 (5.6)	21.25 (1.00)	2.40 (0.88)	0.27 (0.21)
4-6	47.7 (6.7)	21.09 (1.45)	2.69 (1.43)	0.21 (0.11)
6+	45.5 (5.8)	20.28 (1.07)	3.43 (0.98)	0.29 (0.26)

Walking days least 10 min. hours/week^1)^				

1	45.2 (6.4)	21.29 (1.32)	2.40 (1.22)	0.21 (0.18)
2-3	45.2 (7.0)	21.25 (1.23)	2.50 (1.20)	0.25 (0.19)
4-6	48.4 (6.9)	20.59 (1.31)	2.87 (1.14)	0.55 (0.38)
6+	47.6 (5.4)	20.25 (1.31)	3.48 (1.39)	0.28 (0.17)

Sitting hours/week^1)^				

3	45.7 (5.8)	20.38 (1.31)	3.22 (1.27)	0.23 (0.22)
4–6	46.1 (6.3)	20.80 (1.38)	2.88 (1.32)	0.27 (0.23)
7–12	47.2 (5.8)	21.59 (1.10)	2.07 (0.96)	0.27 (0.24)
13+	43.6 (8.0)	22.44 (0.46)	1.36 (0.22)	0.20 (0.27)

^1) ^see  for full text

^2) ^METs = average hours per day.

To further assess validity, we examined the correlation of the self-reported IPAQ responses with VO_2max _and with selected measures from ActiReg. Spearman correlation coefficients are presented in Table 5. For VO_2max_, the correlation was highest with the total vigorous PA (r = 0.41 p ≤ 0.01), vigorous hour PA per week (0.40 p ≤ 0.01) and vigorous days (0.36 p ≤ 0.01) and IPAQ Mets (0.30 p ≤ 0.01). For EE (MJ), the correlations with the questionnaire responses were positive for IPAQ METs (r = 0.26, p ≤ 0.05) and negative for sitting hours per week (r = – 0.25, p ≤ 0.05). Sitting hours per week was also significantly negative with PAL (r = -0.35, p ≤ 0.01) as well as with METs 1–3 (r = 0.44, p ≤ 0.01) and METs 3–6 (r = -0.42, p ≤ 0.01).

**Table 5:** Spearman correlation coefficients for IPAQ (short version, last 7 days) with the VO_2max _and ActiReg to assess criterion validity

Variables						
Measures	VO_2max_	EE(MJ)^1)^	PAL^2)^	METs^3)^1–3	METs^3) ^3–6	METs^3)^6+

IPAQ short version (last 7 days)						

Vigorous days/week	0.36**	0.07	0.08	0.01	0.03	0.09
Vigorous hours/day	0.40**	0.05	0.09	-0.04	0.09	0.05
Total IPAQ vigorous	0.41**	0.05	0.08	-0.02	0.08	0.07
						
Moderate days/week	0.15	0.21	0.17	-0.13	0.16	0.14
Moderate hours/day	0.18	0.17	0.11	-0.05	0.12	0.07
Total IPAQ moderate	0.19	0.16	0.14	-0.1	0.17	0.1
						
Walking days/week	0.18	0.05	0.08	-0.07	0.07	0.19
Walking hours/day	0.14	0.17	0.2	-0.17	0.17	0.23*
Total IPAQ walking	0.14	0.16	0.23*	-0.27*	0.26*	0.24*
						
Total IPAQ sitting	0.07	-0.25*	-0.35**	0.44**	-0.42**	0.31
						
IPAQ Mets min/week	0.30**	0.26*	0.29*	-0.32**	0.34**	0.15
						
3 PA categories	0.31**	0.19	0.24*	-0.31**	0.32**	0.16
						
BMI 3 categories	-0.42**	0.31**	0.45	-0.09	0.08	-0.08

* p < .05, ** p < .01.

^1) ^average EE (MJ) per day

^2) ^PAL = average physical activity level in 7 days

^3) ^METs = average hours per day.

see  for full text

3 PA categories = classification of physical activity in three levels; "low" "moderate" and "high"

BMI 3 categories = Classification of BMI in three levels; "normal" (< 25 kg/m^2^), "overweight" (25–29.99) and "obese" (≥30 kg/m^2^)

The time spent in activities with METs values 6+ based on ActiReg most strongly correlated with hours walking per week as measured with IPAQ (r = 0.23, p ≤ 0.05) and total walking correlated most strongly with METs 1–3, METs 3–6 and METs 6+ (respectively r = -0.27, 0.26, 0.24, p ≤ 0.05) and most weakly with vigorous hours per day (r = 0.05). IPAQ METs correlated most strongly with METs 3–6 and METs 1–3 from ActiReg (r = 0.34 and -0.32, p ≤ 0.01). Classification of PA in three levels correlated also most strongly with VO_2max _(0.31 p ≤ 0.01) and METs 3–6 and METs 1–3 from ActiReg (r = 0.32 and -0.31, p ≤ 0.01). Classification of BMI in three levels correlated most strongly negative with VO_2max _(-0.42 p ≤ 0.01) and MJ from ActiReg (r = 0.31 p ≤ 0.01).

## Discussion

In this study of young adult men, we found evidence for good reliability with high correlations between the test – retest for the IPAQ questionnaire for vigorous days, hours and sitting hours per day and moderate for walking days and hours. Reliability was further fair for moderate activity days and hours.

Concerning validity, our results suggest that total IPAQ vigorous PA, was a moderately good measure of vigorous activity, having moderately strong, significant correlations with VO_2max_, but correlated weakly with METs values of 6 or more measured with ActiReg. Only the IPAQ for walking had a fair correlation with METs 6+. The IPAQ sitting hours per week was moderately correlated with METs values of 1–3 and negatively correlated with METs values of 3–6.

### Reliability

Our study provides useful information about the reproducibility of the IPAQ short version. The reliability ranged from good for sitting and vigorous PA, and moderate for walking, to fair for moderate activity. Brown et al. [21] assessed test-retest reliability of four PA measures used in population studies and one of them was the short IPAQ. They pointed out that the ICC was lowest for the moderate questions (0.16 to 0.44). In our study we also have the lowest ICC for the moderate questions (0.16–0.51 and 0.09–0.49). Since the development of the IPAQ, the EUPASS and the WHO have used it for monitoring and surveillance. However, some have raised concern that use of the IPAQ may be associated with over-reporting of PA. In one study, 75% of subjects reported less PA with the modified procedure than with the short IPAQ telephone survey [3]. Twenty three of the 50 individuals were found to have reported some amounts of PA with IPAQ (either walking, or vigorous or moderate PA) when they should have reported none. The authors discuss how the IPAQ protocol asks respondents to report average time per day in each intensity category. In this way if PA is reported for more than a day, the respondents may report an average time per day and could over-report the mean time per day, by reporting the day they were most active. Another study gives some indirect support to overestimation of habitual PA in obese [22]. In the EUPASS study [5] the test-retest reliability scores of the IPAQ (short version, last 7 days telephone interview, S7T), were rather low. They concluded that more research was needed to further investigate and improve the quality of IPAQ.

Our results for re-test reliability of the IPAQ short version were good for vigorous and fair for moderate activities. The reliability of the IPAQ S7T from eight participating EU countries in general ranged from 0.3 and 0.5, which appears to be rather low for reliability. In the Chinese version of IPAQ, the test-retest reliability was completed twice with a three-day interval among college students and validity by Caltrac accelerometer [6]. The short IPAQ had ICC above 0.7 for PA. They concluded that both the long and short version had acceptable reliability and validity, compared to other PA instruments. However, the short version, underestimated the EE of total and moderate PA.

The IPAQ was developed to overcome differences in PA measurement. However, the IPAQ is itself a new instrument, making it difficult to compare it with other, older questionnaires. Many other measurement studies focus mainly on leisure time PA (LTPA). The study of Rütten et al. [4] highlights some of the methodological innovations of the IPAQ instrument. One is that many measurements methods focus mainly on LTPA, while the IPAQ integrates several domains and not only PA at work. This methodological shift may explain some of the differences i.e. in overall scores for PA.

### Validity

#### IPAQ and VO_2max_

Assessment of criterion validity depends on the validation criteria. In our study VO_2max _was chosen as the primary criterion. The results in this study are similar to those often obtained in the general population with correlation coefficients between PA and fitness typically 0.3–0.5 [23]. In a study that evaluated 10 commonly used PA questionnaires [24], the correlation of VO_2max _was stronger with reported heavy activity than for light activity. Fogelholm et al. [9] validated IPAQ against fitness in men aged 21–43 years. They found that a weekly frequency of vigorous PA showed positive association with fitness. Fogelholm found mostly similar characteristics of young men (aged 21–43) in their study as in ours (aged 20–39, Table 1). In our study total vigorous PA, hours per week and days were most strongly correlated (respectively 0.41, 0.40 and 0.36, r= p ≤ 0.01) with VO_2max_.

#### IPAQ and ActiReg

To evaluate validity further we also used several directly measured aspects of PA measures assessed with ActiReg, including EE, PAL and METs 1–3. METs 3–6 and METs 6+. METs values of 6 or more measured with ActiReg correlated not, except for total IPAQ walking and walking hours per week, which correlated fair with METs 6+. Sitting hours per week correlated moderately with METs values of 1–3 (0.44) and negatively correlated with METs values of 3–6 (-0.42). Ekelund et al. [10] indicated moderate criterion validity of the IPAQ instrument and the MTI Actigraph (Manufacturing Technology Inc., formerly known as The Computer Science and Application, (CSA)) with correlation ranging from 0.16–0.35. Craig et al. obtained the same correlation in their study [1]. In the Chinese version of IPAQ [8] using an MTI accelerometer there was weak agreement between IPAQ and the total MTI – derived activity and sub-components. In our study there is an average underestimation of IPAQ (total moderate, vigorous and walking) with -433 min/week, limits of agreement 1605 to – 2471 min/week compared to ActiReg calculated in a Bland Altman plot (plot not shown). We think that the main reason for the underestimation by IPAQ is due to the fact that the questionnaire only asks for time periods with duration of 10 min and more, while ActiReg register each minute of the day with sufficient PA. The Chinese short version of IPAQ [6] also underestimated EE of total and moderate PA. Also Hallal & Victora [7] reported possible underestimation of PA levels.

#### IPAQ and total time of PA expenditure

The IPAQ especially focuses on investigation of total time of PA energy expenditure (MET min ^-1^) as these PA indicators are associated with reduced cardiovascular diseases and mortality and therefore have been applied in public health recommendations [25]. Rütten et al. [4] compared IPAQ results with other European studies and PA in the IPAQ-S7T and Germany had the highest median of vigorous PA, 26 min ^-1^. In our study we had a mean of 16.11 average minutes per day or 0.27 hours per day assessed with ActiReg and METs 6+. In the study of Ekelund et al. [10] total self-reported PA (MET-min day ^-1^) was significantly correlated with average intensity of activity (counts min ^-1^) from accelerometry (r = 0.34, p < 0.001). Further in our study IPAQ METs correlated most strongly with METs 3–6 and METs 1–3 measured with ActiReg (r = 0.34 and -.032, p ≤ 0.01) and VO_2max _(r = 0.30, p ≤ 0.01) and more weakly with total EE (MJ) and PAL (r = 0.26 and 0.29, p ≤ 0.05).

In our study the correlation between vigorous activity and VO_2max _are quite good, but vigorous activity do not correlate wit ActiReg 6+. This stronger association with the measures of vigorous activity is consistent with the pattern found in comparisons using VO_2max_. The fair correlations with weekly hours of time spent at > 6 METs measured with ActiReg and the weaker association with other measures as with EE and PAL is not surprising. PAL is primarily determined by time spent in activities with low and moderate intensities, and that high intensity activity has little impact on daily EE [26]. Probably this also is the answer to why our result of walking correlates much better with ActiReg than ActiReg and METs 6+. Our results suggest that IPAQ S7S was a better measure of intense PA, rather than EE. The study of criterion validity of IPAQ short version in Swedish adults [10] showed that self-reported time in PA was significantly different from time measured by accelerometry.

Strengths of our study are use of two objective validation measures, such as use of treadmill derived maximal oxygen uptake values and ActiReg measuring PA and EE during waking hours across a 7-day observation interval for validation, and a one-week test-retest repeatability design. Study strengths are also the random selection of study participants from a larger existing study population. Previous studies usually evaluating the validity of PA questionnaire often include selected samples of volunteers, also IPAQ itself [1]. The limitation is however, due to economic resources at that time, a study of only men and the low response-rate may affect the results because the likelihood of bias. The age ranges is also narrow, which limits the application of results to older men.

## Conclusion

In conclusion, our results indicate that the IPAQ short version for men has acceptable test-retest reliability and criterion validity for vigorous activity and sitting. Walking has moderate reliability. Only the IPAQ for walking had a fair correlation with METs 6+. The IPAQ sitting hours per week was moderately correlated with METs values of 1–3 and negatively correlated with METs values of 3–6. The questions about moderate activity had fair reproducibility and correlated poorly with most comparison measures.

## Competing interests

The authors declare that they have no competing interests.

## Authors' contributions

NK was responsible for study design, manuscript preparation, data collection and statistical analysis. VG and B-EH were respectively responsible for VO_2max _testing and ActiReg data. All authors have read and approved the final manuscript.

## Pre-publication history

The pre-publication history for this paper can be accessed here:


